# What is a mental disorder? An exemplar-focused approach

**DOI:** 10.1017/S0033291721001185

**Published:** 2021-04

**Authors:** Dan J. Stein, Andrea C. Palk, Kenneth S. Kendler

**Affiliations:** 1 SAMRC Unit on Risk & Resilience in Mental Disorders, Department of Psychiatry and Neuroscience Institute, University of Cape Town, Cape Town, South Africa; 2 Department of Philosophy, Stellenbosch University, Stellenbosch, South Africa; 3 Virginia Institute of Psychiatric and Behavioral Genetics and Departments of Psychiatry, and Human and Molecular Genetics, School of Medicine/Virginia Commonwealth University, VA, USA

**Keywords:** nosology, categorization, DSM

## Abstract

The question of ‘what is a mental disorder?’ is central to the philosophy of psychiatry, and has crucial practical implications for psychiatric nosology. Rather than approaching the problem in terms of abstractions, we review a series of exemplars – real-world examples of problematic cases that emerged during work on and immediately after DSM-5, with the aim of developing practical guidelines for addressing future proposals. We consider cases where (1) there is harm but no clear dysfunction, (2) there is dysfunction but no clear harm, and (3) there is possible dysfunction and/or harm, but this is controversial for various reasons. We found no specific criteria to determine whether future proposals for new entities should be accepted or rejected; any such proposal will need to be assessed on its particular merits, using practical judgment. Nevertheless, several suggestions for the field emerged. First, while harm is useful for defining mental disorder, some proposed entities may require careful consideration of individual *v.* societal harm, as well as of societal accommodation. Second, while dysfunction is useful for defining mental disorder, the field would benefit from more sharply defined indicators of dysfunction. Third, it would be useful to incorporate evidence of diagnostic validity and clinical utility into the definition of mental disorder, and to further clarify the type and extent of data needed to support such judgments.

## Introduction

The question of ‘what is a mental disorder?’ is foundational in philosophy of psychiatry, and also has enormous practical importance for clinicians and patients. This question has therefore been addressed in successive revisions of the American Psychiatric Association's Diagnostic and Statistical Manual of Mental Disorders (DSM). Given ongoing work on the revision of DSM-5, it is timely to ask this question again.

Many previous attempts have applied a conceptual approach to the definition of mental disorder. These have produced limited progress, particularly in assisting with decisions about specific conditions. Thus, it may be useful to try a different approach to this critical problem. Rather than focusing on abstractions, we review a series of exemplars – real-world examples of problematic cases that emerged during work on and immediately after DSM-5. From these cases, we hoped to extract practical guidelines for considering future proposals for the inclusion of entities in the nosology.

## What is a mental disorder?

The question of ‘what is a mental disorder’, is crucial, in part, because the real possibility exists of erroneously classifying various kinds of social deviance or behavioral variation as ‘disorder’, when they are better conceptualized using other categories, such as ‘non-pathological individual differences’, ‘lifestyle choice’, or ‘crime’. A paradigmatic example from DSM is that of homosexuality, which was conceptualized in DSM-I as a disorder, (American Psychiatric Association, 1952) but by DSM-5 was no longer mentioned (American Psychiatric Association, 2013; Drescher, 2015).

Many authors have emphasized that what counts as a disease or disorder changes over time and across place, and have accused medicine and psychiatry of failing to recognize how idioms of distress are shaped by culture (Kirmayer, 2005; Kleinman, 1988). Others have accused the DSM of over-medicalizing (Frances, 2014; Horwitz, 2007; Szasz, 2007). These criticisms are driven by disagreements about the advantages and disadvantages of the medicalization of putative mental conditions. Central to these debates is the degree to which our disorders can be best understood as independent biological entities (naturalism/objectivism) or value-laden social constructs (normativism/constructivism) (Agich, 1983; Boorse, 1975; Fulford, 2001; Nordenfelt, 2007; Sadler, 2005; Stein, 2008; Zachar & Kendler, 2017).

Prior proposals have attempted to move beyond the polarities of naturalism and constructivism. Zachar suggested that mental disorders are ‘practical kinds’ (Zachar, 2002), shifting the issue from whether disorder categories reference scientific entities, to how effectively they facilitate particular scientific or clinical goals (Zachar & Kendler, 2017). In influential work, Wakefield defined mental disorders as ‘harmful dysfunctions’, and depicted dysfunction in evolutionary terms (Wakefield, 1992).

A strong form of realism holds that, just as the periodic table depicts the properties of molecular entities, so a medical or psychiatric nosology can carve nature at its joints – as a series of ‘natural kinds’ (Kendler, 2016; Stein, 2008). Softer forms of realism, likely more appropriate for conceptualizing mental disorders, regard exemplars like biological species as more appropriate for psychiatric disorders as the boundaries between different species are fuzzy, and not amenable to depiction in tabular format (Kendler, 2016; Stein, 2008).

We find aspects of both pragmatic approaches and Wakefield's characterization helpful, and use them as a framework for organizing our exemplars. More specifically, in reviewing real-world cases relevant to DSM-5 we will rely on the notions of ‘harm’ and of ‘dysfunction’. Harm may be indexed by the presence of distress and impairment, while dysfunction may be inferred when psychobiological mechanisms produce symptoms and associated harm. Nevertheless, as our exemplars will demonstrate, judgments about harm and dysfunction entail a range of additional complex considerations.

## DSM definitions of mental disorder

DSM has responded to these debates in its definitions of mental disorders. Thus DSM-III emphasizes, for example, that clinicians should not misclassify or label a cultural expression of distress or political deviance as a disease (American Psychiatric Association, 1980). Subsequent editions of DSM have emphasized that the boundaries of mental disorders are fuzzy (Table 1) (American Psychiatric Association, 2000, 2013).

**Table 1.** DSM-IV definition of mental disorder

Features	
A	A clinically significant behavioral or psychological syndrome or pattern that occurs in an individual.
B	Associated with present distress (e.g. a painful symptom) or disability (i.e. impairment in one or more important areas of functioning) or with a significantly increased risk of suffering death, pain, disability, or an important loss of freedom.
C	Must not be merely an expectable and culturally sanctioned response to a particular event (e.g. the death of a loved one).
D	A manifestation of behavioral, psychological, or biological dysfunction in the individual.
E	Neither deviant behavior (e.g. political, religious, or sexual) nor conflicts that are primarily between the individual and society are mental disorders unless the deviance or conflict is a symptom of a dysfunction in the individual.
Other considerations	
F	No definition adequately specifies precise boundaries for the concept of ‘mental disorder’.
G	The concept of mental disorder (like many other concepts in medicine and science) lacks a consistent operational definition that covers all situations.

During the development of DSM-5, along with others, we attempted to further clarify the DSM criteria for a mental disorder (Table 2) (Stein et al., 2010). While our proposal differs modestly from the later DSM-5 wording (Table 3), three differences are relevant here. First, while the DSM-5 definition refers to dysfunction in ‘psychological, biological, or developmental processes,’ we prefer ‘psychobiological’, to emphasize that psychology and biology are intertwined constructs that encompass development, as well as other life-course constructs.

**Table 2.** DSM-V proposal for the definition of mental/psychiatric disorder

Features	
A	A behavioral or psychological syndrome or pattern that occurs in an individual
B	The consequences of which are clinically significant distresses (e.g. a painful symptom), or disability (i.e. impairment in one or more important areas of functioning)
C	Must not be merely an expectable response to common stressors and losses (e.g. the loss of a loved one) or a culturally sanctioned response to a particular event (e.g. trance states in religious rituals)
D	That reflects an underlying psychobiological dysfunction.
E	That is not primarily a result of social deviance or conflicts with society
Other considerations	
F	That has diagnostic validity on the basis of various diagnostic validators (e.g. prognostic significance, psychobiological disruption, response to treatment)
G	That has clinical utility (e.g. contributes to better conceptualization of diagnoses, or to better assessment and treatment)
H	No definition perfectly specifies precise boundaries for the concept of either ‘medical disorder’ or ‘mental/psychiatric disorder’
I	Diagnostic validators and clinical utility should help to differentiate a disorder from diagnostic ‘nearest neighbors’
J	When considering whether to add a mental/psychiatric condition to the nomenclature or delete a mental/psychiatric condition from the nomenclature, potential benefits (e.g. provide better patient care, stimulate new research) should outweigh potential harms (e.g. hurt particular individuals, be subject to misuse)

**Table 3.** DSM-5 definition of mental disorder

A mental disorder is a syndrome characterized by clinically significant disturbance in an individual's cognition, emotion regulation, or behavior that reflects a dysfunction in the psychological, biological, or development processes underlying mental functioning. Mental disorders are usually associated with significant distress or disability in social, occupational, or other important activities. An expectable or culturally approved response to a common stressor or loss, such as the death of a loved one, is not a mental disorder. Socially deviant behavior (e.g. political, religious, or sexual) and conflicts that are primarily between the individual and society are not mental disorders unless the deviance or conflict results from a dysfunction in the individual, as described above.

Second, our proposal suggested that the consequences of a mental disorder are clinically significant distress or disability (B). The DSM-5 wording indicates that mental disorders are *usually* associated with significant distress or impairment. The word ‘usually’ may be technically accurate, in that on rare occasions, a mental disorder is listed in DSM-5, and there is no ‘clinical criterion’ (First & Wakefield, 2013). However, given that psychiatric symptoms are often on a continuum with normality, the clinical criterion is one key way of providing a relatively valid and reliable marker of underlying dysfunction, so lessening the risk of false positives and over-medicalization (Cooper, 2013). Other ways in which clinical criteria can validly and reliably point to underlying dysfunction include descriptions of symptom severity, excessiveness, frequency, and duration (First & Wakefield, 2013).

Third, our proposal made reference to considerations of diagnostic validity and clinical utility. This explicitly emphasizes that decisions about proposals for new entities must address empirical data. Certainly, data on diagnostic validity and clinical utility of proposed entities were carefully assessed during the DSM-5 revision process.

## Examining different exemplars

We now turn to a number of test-cases that emerged during DSM-5. While conceptual work is crucial, it is important to examine its conclusions in the context of specific empirical examples, which may then produce greater clarity on the underlying conceptual issues.

We explore, in turn, several different types of cases, categorized along the following lines: (1) entities associated with harm, but for which there is limited evidence of underlying dysfunction, (2) entities involving dysfunction but without strong evidence that they produce harm, and (3) entities involving possible harm and dysfunction, and thus possibly indicative of a disorder, but which are controversial for various reasons. While the third category deals explicitly with controversial cases, controversy is present in all three categories.

### Harm but no clear psychobiological dysfunction

A number of conditions are associated with harm to individuals and/or society, but are not considered disorders because they lack evidence of underlying psychobiological dysfunction. Entities that fall under this rubric include unwanted physical, mental, or behavioral changes (e.g. those that accompany aging), more enduring traits that entail suffering or produce negative impacts but are not considered disorders (e.g. laziness), and behavior that is more appropriately classified as culturally or socially deviant rather than as a mental disorder (e.g. racism). The appropriate responses to distress or impairment associated with these entities would generally be regarded as emanating from moral, cultural, or social domains, rather than from the domain of health. Closer examination of specific exemplars suggests, however, that judgments of whether or not an entity should be included in the nosology reflect a number of different considerations (Table 4).

**Table 4.** Key considerations regarding the inclusion of putative entities in the nosology

Typology of disorders	Exemplars	Key considerations
Harm but no clear psychobiological dysfunction	Aging	Existence, efficacy, and cost-efficiency of health interventions
	Bereavement exclusion criterion	Internal consistency of criteria and constructs in the nosology
	Racism	Relevance of social and cultural values and interventions
	Laziness/apathy, gluttony/hyperphagia, acquisitiveness/hoarding, etc.	Presence of associated features, including severity, that indicate dysfunction
Psychobiological dysfunction but no clear harm	Auditory hallucinations	Extent of distress and impairment indicative of harm
	ASD	Potential for social accommodation to diminish harm
	GD	Weighing the advantages/disadvantages of medicalization.
Possible harm and psychobiological dysfunction but controversial Medicalization concerns	Compulsive sexual behavior disorder, Gaming disorder	Assessment of degree of loss of control, and associated impairment
Overdiagnosis concerns	APS	Sufficient data to assess advantages/disadvantages of health interventions
	Suicidal behavior	Self-harming behavior is not necessarily indicative of a mental disorder
Pragmatic concerns	Simple type schizophrenia	Rare and poorly researched entities may be disorders, but may not deserve inclusion in the nosology
	PCD	Maintaining societal trust in the integrity of psychiatric diagnosis
	PMDD	Weighing responsibilities to patients *v.* responsibilities to society

Aging is associated with a range of negative sequelae. Furthermore, there is a growing understanding of the specific psychobiological mechanisms that lead to symptoms associated with aging and these harms, bolstering the claim that aging involves dysfunction (De Grey, 2007). That said, a range of causal mechanisms presumably underly the spectrum of aging from premature aging (e.g. progeria) to typical senescence. Indeed, a view that emphasizes the normality of aging may concede that physicians counsel individuals on a range of measures to sustain health and curb aging but call into question the inclusion of mild neurocognitive disorder in DSM-5. The concern is that this risks pathologizing minor forgetfulness associated with the aging process, particularly given the lack of treatment and the potentially harmful effects of receiving such a diagnosis (Rattan, 2014). That said, the more future medical interventions for mild neurocognitive disorder target mechanisms relevant to premature aging, and are shown efficacious and cost-effective, the more useful such a diagnosis will, arguably, be. Thus, judgments about the inclusion of entities in the nosology may, in part, reflect the existence, efficacy, and cost-efficiency of health interventions.

Time-limited and non-incapacitating anxiety associated with threat (e.g. a possible job loss), and suffering associated with loss (e.g. death of a parent), may be experienced as unwelcome, and clinicians may play a useful role in helping to alleviate them. Nevertheless, anxiety and sadness in the face of threat and loss are generally considered to be appropriate, rather than dysfunctional, responses. During the development of DSM-5, there was considerable debate about the removal of the bereavement exclusion criteria from the diagnosis of major depression (Zachar, First, & Kendler, 2017). The removal of this clause is consistent with the fact that depressions that are precipitated by a range of other common stressors (e.g. romantic rejection; serious medical problems) were not excluded. While critics argued that this decision reflected over-medicalization, a counter-argument is that it is important to ensure that diagnostic criteria allow appropriate diagnosis and treatment of depression in the context of bereavement (Prigerson, Boelen, Xu, Smith, & Maciejewski, 2021). Thus, judgments about thresholds for a putative disorder in the nosology may require consideration of epistemic values such as the internal consistency of criteria.

Racism is a phenomenon that has been associated with great harm and suffering (Schmitt, Branscombe, Postmes, & Garcia, 2014). While extreme racism may be a symptom of psychopathology, and there is some evidence of an association between, for example, racism and certain personality types (Adorno, 1969), there is little evidence that racism, in general, is the result of underlying psychobiological dysfunction. Rather, there is relatively widespread consensus that racist beliefs and behavior are largely a product of socialization and culture. We would therefore argue that racism is not a disorder; it is a phenomenon that, while sanctioned in some cultures in the past, is now a form of social deviance that should be addressed by a range of different social and educational interventions. Thus, judgments about the inclusion of an entity in the nosology may require rigorous reflection on cultural and social values.

Similar logic would hold for a range of other socially deviant or problematic behaviors (Aristotle, 1985), including those redolent of the seven deadly sins of laziness, gluttony, acquisitiveness, aggression, lust, jealousy, and pride. Prima facie, these are more appropriately understood and responded to in moral or socio-cultural terms rather than with health interventions. That said, psychotherapy may usefully target such behaviors or traits, and public health may usefully advocate for healthy eating and sexual behaviors. Furthermore, this matter is complicated by the fact that when clearly excessive, such traits can point to underlying psychobiological dysfunction; indeed, symptoms such as apathy, hyperphagia, hoarding, violence, hypersexuality, obsessional jealousy, and grandiosity may be indicative of a psychiatric disorder, and are appropriately listed in the DSM-5 glossary. Thus, judgments about the inclusion of a disorder in the nosology are based, in part, on evidence of clear excessiveness of behaviors/traits, and associated features that point to dysfunction.

### Psychobiological dysfunction but no clear harm

In this category, we include various conditions for which there is some evidence of underlying psychobiological dysfunction, even if this is not fully understood. Conditions in this category may have been regarded as harmful, in the sense of disadvantageous, or socially deviant, in the past, but this view has been contested due to social change. While conditions in this category may point to differences rather than disorder, individuals with these conditions may still experience disadvantage and suffering. It may therefore be crucial to ensure support and treatment for those who seek it. Again, a closer examination of specific exemplars suggests that judgments of whether or not an entity should be included in the nosology reflect a number of different considerations (Table 4).

The notion of disability has been extensively challenged by rights-based advocacy groups and organizations that have focused on promoting inclusivity, equality, and respect (Charlton, 1998). A paradigmatic example is deafness, which although not a psychiatric entity, is nevertheless useful as a point of departure for further discussion of analogous behavioral conditions where the presence of harm is contested. Deafness is the result of underlying alterations in structures and mechanisms of hearing, consistent with dysfunction. Moreover, given the challenges of participating in a hearing society, deafness has been widely viewed as disadvantageous, and characterized as a medical condition. However, this has been challenged by the view that deafness itself is not intrinsically harmful; rather, it is societal responses, or lack of response in terms of ensuring adequate accommodation, that produces harm. A view of deafness as a disability has been replaced with a view of deafness as a cultural identity (Padden & Humphries, 2005). This identity is referred to as Deaf, rather than deaf, which refers simply to hearing loss. While there have been rare, but controversial, cases of Deaf parents wishing to utilize preimplantation genetic diagnosis to select for deafness, many members of the Deaf community, given the choice of having children with or without hearing, opt for the former (Camporesi, 2010; Wallis, 2020).

Deaf culture has some parallels with groups that are open about their unusual psychological behaviors or traits, but who argue that these are not associated with harm. It turns out, for example, that hearing voice is prevalent in the general population, and that these experiences may not necessarily be indicative of a serious mental disorder (Maijer, Begemann, Palmen, Leucht, & Sommer, 2018). In the absence of harm, it is difficult to argue for the medicalization of such experiences, and there are now support groups for those with these experiences (Longden, 2017). That said, hearing voices may be a symptom of a range of mental disorders, other than psychotic disorders, and there is evidence from community surveys that such symptoms are associated with significant disability, which is unlikely to be simply a reflection of lack of social accommodation (Navarro-Mateu et al., 2017; Pierre, 2010). Thus, judgments about whether or not an entity should be included in the nosology require nuanced assessment of the extent of harm, as reflected in distress and impairment.

Autistic spectrum disorder (ASD) which is associated with alterations in structures and mechanisms underlying behavior (Van Rooij et al., 2018), has traditionally been viewed as a harmful condition. However, there is a contrary position, which may be particularly relevant to milder cases of ASD. In this view, the positive attributes associated with ASD (e.g. high levels of creativity and mathematical ability) are emphasized and neurodiversity is celebrated, shifting the onus onto neuro-typical society to accommodate neuro-atypical persons (Glannon, 2007). However, despite the growing prevalence of persons with ASD who choose to see themselves as situated on a spectrum of normal variation, there are many individuals and families who seek health interventions or advocate for more scientific research to cure or prevent ASD (Walsh, Elsabbagh, Bolton, & Singh, 2011). These disagreements are perhaps indicative of the heterogeneous and dimensional nature of both ASD and its impact; in severe cases care rather than accommodation is required. Thus, judgments about whether or not an entity should be included in the nosology require careful assessment of the extent to which social accommodation is possible.

A similar set of issues emerges for gender identity disorder (GID) or transsexualism, which were removed from DSM-5 and ICD-11 and replaced by gender dysphoria (GD) and gender incongruence, respectively. These latter categories address cases in which there is significant distress due to conflicts between assigned and identified gender. In the case of GD, there is some preliminary evidence of neuroanatomical differences between transgender and cisgender persons which may arguably indicate underlying dysfunction (Burke, Manzouri, & Savic, 2017). Moreover, there is also some evidence of harmfulness, for example, a high risk of suicide (Garcia-Vega, Camero, Fernandez, & Villaverde, 2018). This could be sufficient for inclusion in our third category, however, we mention GD here because, despite the evidence that distress is intrinsic to the condition, it has also been argued that this distress is a product of stigmatization and social rejection. The shift from social rejection to acceptance of homosexuality, has bolstered this argument for some. On the other hand, from a clinical utility perspective, the inclusion of GD in the nosology is precisely important for ensuring medical and psychiatric care for individuals with this condition who request such care. Judgments about whether or not an entity should be included in the nosology may require careful balancing of the advantages and disadvantages of medicalization (Parens, 2013).

### Possible harm and psychobiological dysfunction, but controversial

In the third category, we include conditions for which there is some evidence of underlying psychobiological dysfunction and actual or potential harm, but which are controversial for various reasons. First, the controversy may be attributed to a lack of certainty about whether or not a condition does, in fact, reflect underlying psychobiological dysfunction, or whether inclusion would represent over-medicalization. Second, the controversy could arise due to the fact that harm, in the sense of clinically significant distress or impairment, may be present only as a risk, which may not be actualized, so that inclusion of the condition may lead to overdiagnosis. Concerns about medicalization and overdiagnosis both reflect a critical stance towards the expansion of disorder constructs (Hofmann, 2016). Third, a condition may be indicative of disorder but considered controversial, in the sense of inappropriate for inclusion in the nosology, due to various pragmatic concerns. This could include a risk of misuse in legal contexts or negative implications for public health. These kinds of pragmatic considerations shift the focus from whether or not a condition is a disorder to whether or not a particular disorder belongs in a diagnostic manual (Table 4).

#### Medicalization concerns

Compulsive sexual behavior disorder was rejected for DSM-5 but is included in ICD-11 as an impulse control disorder (Grant & Chamberlain, 2016). There is a growing evidence base on this disorder. Still, hypersexuality is not necessarily pathological, and there is currently little direct evidence that those who present clinically for the treatment of compulsive sexual behavior have underlying psychobiological dysfunction. Thus, such dysfunction needs to be inferred on the basis of clinical criteria such as severity and duration of symptoms (Kafka, 2010). As noted earlier, psychiatry should be wary of medicalizing conditions redolent of the seven sins, focusing rather on advocating for healthy sexual behavior. At the same time, psychiatry clearly has a role when hypersexuality reflects an underlying medical or psychiatric disorder, and it may well have a role when symptoms are truly excessive and associated with a great deal of distress and impairment. For example, it is not clear whether a person who compulsively watches pornography, but is able to limit viewing to the privacy of the home, has a disorder. While personal relationships may be negatively impacted, such a person can be described as functioning, as long as there is control over the behavior. We would be more inclined to regard a person who cannot limit viewing of pornography to a particular time of day or place and feels compelled to watch it while at work, with risk of job loss, as having a disorder. Judgments about whether or not an entity should be included in the nosology may require careful assessment of the degree of loss of control, and related impairment, particularly in the case of compulsive or addictive behaviors.

Internet gaming disorder was included in DSM-5 as a condition for further study, and gaming disorder is included as a mental disorder in ICD-11 (Billieux, Flayelle, Rumpf, & Stein, 2019). There is some evidence of underlying alterations in psychobiological structures and mechanisms in gambling disorder, which is included in both nosologies, but less evidence that this is the case in gaming disorder. Behavioral addictions are controversial partly because they raise questions as to whether underlying alterations in structures or mechanisms are sufficient to explain the behavior (which may be viewed as a lifestyle choice rather than as a loss of control). Proposals for new behavioral addictions such as gaming disorder also face the difficulty that there is simply less evidence for newly emergent conditions. Similarly, the brain disease model of substance use disorders has been critiqued (Hammer et al., 2013). Still, there is a strong argument that substance use disorders are mental disorders, with evidence of alterations in a range of psychobiological processes that are associated with loss of control, and that can be targeted by health interventions.

#### Overdiagnosis concerns

Attenuated psychosis syndrome (APS), which is associated both with evidence of psychobiological dysfunction and potential harm in the case of conversion, was included in DSM-5 as a condition for further study (Tsuang et al., 2013). APS elicits concerns about overdiagnosis, mainly due to the possibility that interventions for individuals who meet the criteria may cause harm (Zachar, First, & Kendler, 2020). There are some parallels between APS and other risk-syndromes such as hypercholesterolaemia or hypertension. Once it was clear that high levels of cholesterol were risky, these were defined as pathological. With the introduction of statins, and evidence that these agents lowered risks, thresholds for diagnosis were lowered; with the introduction of generic statins, and great cost-efficiencies, such thresholds were further decreased. It is possible that an analogous perspective may be useful in defining thresholds for anxiety disorders and depression. However, in the case of APS, there are arguably insufficient data demonstrating risk if untreated, as well as insufficient data demonstrating safety, efficacy, and cost-efficiency of interventions. Moreover, medical risk-syndromes may differ from the risk associated with a psychotic disorder due to the high levels of stigmatization associated with the latter. Nevertheless, it is possible that the issue of whether, and when, to intervene in the case of evidence of psychiatric risk will become increasingly pertinent given the potential for identifying predictive biomarkers – for example, from molecular genetics research (Palk, Dalvie, de Vries, Martin, & Stein, 2019).

Suicidal behavior disorder is included in DSM-5 as a condition for further study. Clearly, it is important for clinicians to be aware of suicidal behavior, and this is often an important target of treatment. On the other hand, suicidal behavior may be due to a range of different mental disorders, reflecting a range of different kinds of dysfunction. Furthermore, suicidal behavior is not always associated with a mental disorder; there is a compelling argument that in particular medical circumstances, it is understandable and appropriate for patients to make a decision to end their lives. Suicide can also arise as a form of political protest or a culturally sanctioned response to shame. Judgments about diagnostic validity may be complex, including consideration of a range of different empirical data of varying quality. This point is also exemplified by other entities included in DSM-5 as conditions for further study, namely persistent complex bereavement disorder, depressive episodes with short-duration hypomania, caffeine use disorder, non-suicidal self-injury, and neurobehavioral disorder associated with prenatal alcohol exposure (American Psychiatric Association, 2013).

#### Pragmatic concerns

Simple (type) schizophrenia (SS) or simple deteriorative disorder has long been controversial (Serra-Mestres et al., 2000). It has not been included in the nosology since DSM-III (although it was included in DSM-IV as a condition for further study), and while it was in ICD-10 it is not in ICD-11. There is indeed some evidence that simple schizophrenia is a rare deteriorative disorder characterized by nonspecific negative symptoms and an absence of psychotic symptoms. However, while previous iterations of DSM contained schizophrenia sub-types, these were appropriately removed due to a lack of diagnostic validity and reliability, and evidence that schizophrenia is a spectrum disorder (Serra-Mestres et al., 2000; Whitwell, Bramham, & Moriarty, 2018). Nevertheless, the fact that there continue to be patients who present with these kinds of deteriorative symptoms has been used to support claims that the diagnosis remains relevant (Whitwell et al., 2018). This exemplar illustrates that there is a distinction between judgments regarding whether a condition is a mental disorder, and judgments regarding whether it should be included in the nosology.

Paraphilic coercive disorder (PCD) was considered, but ultimately rejected, for inclusion in DSM-5 (Stern, 2010). PCD illustrates issues at the boundary between the medical and legal systems, and highlights disagreements about the nature of psychopathology and moral responsibility. There is inconclusive evidence of underlying psychobiological dysfunction or of harm to the individual (other than that following legal transgression) (Knight, 2010). However, more relevant here is the real risk of the PCD diagnosis being misused in legal contexts to either inappropriately exculpate a rapist, or to detain persons indefinitely, if deemed to be at risk of sexual reoffending (Wakefield, 2011). The debates surrounding PCD highlight how pragmatic considerations inform decisions about nosology. Such considerations include maintaining societal trust in the integrity of psychiatric diagnosis and protecting the reputation of the profession, as well as anticipating potentially harmful consequences of including certain constructs as disorders.

Importantly, as social mores change, so too may considerations about the cost-benefit of including particular entities in the nosology. Premenstrual dysphoric disorder (PMDD), formerly known as late luteal phase dysphoric disorder, is well described in the psychiatric literature. There is clear evidence that specific psychobiological mechanisms are altered in those with this condition, and that those with this condition may benefit from medical treatments (Epperson et al., 2012). Still, this entity was not included in DSM-IV, as concerns were raised that the diagnosis would impact negatively on women, confirming stereotypes that they had less ability to fulfil professional obligations (Zachar & Kendler, 2015). In DSM-5, perhaps partly because of advances in our understanding of and treatment of PMDD, and perhaps partly because of continued advances in gender parity, PMDD was included in the manual. Judgments about the inclusion of entities in the nosology may need to weigh up responsibilities to patients *v.* responsibilities to society as a whole.

## Discussion

Taken together, these exemplars may help shed light on key conceptual issues involved in including a proposed entity in the classification.

One set of conceptual issues surround the notion of ‘harm’. Harm refers to suffering or disadvantage associated with a particular condition, and is operationalized with the ‘clinical criterion’ of DSM-5 using the phrase ‘significant distress and/or impairment’. It has often been emphasized, including by DSM-5, that this criterion is ‘fuzzy’, and also that not all distress/impairment points to a mental disorder. However, our exemplars indicate a number of additional complexities.

First, decisions about the introduction of new entities into the nosology need to balance the harm to the individual with harm to society. This is seen in the discussion of PCD and PMDD. The introduction of PCD has significant potential for societal harm, and the proposal to introduce this disorder was rejected. While there were concerns about such harm for PMDD, societal changes have significantly mitigated these concerns, and the proposal to introduce this disorder was accepted. Furthermore, putative PCDs are relatively rare and PMDD relatively common, so the possibility of clinical benefit to those affected is greater for the latter (Hartlage, Breaux, & Yonkers, 2014; Robinson & Ismail, 2015; Thornton, 2010; Wollert, 2011). Second, there may be significant debate about the extent to which harm is due to the failure of society to accommodate differences. This is seen in debates around the inclusion of homosexual and gender dysphoria in the nosology. In the former case, exclusion was agreed upon, while in the latter case inclusion was advocated.

While the concept of ‘harm’ is a useful one for defining mental disorder, when new entities are proposed in the future, it will be important to consider, for some of them, more sharply, the issue of individual *v.* societal harm, as well as the issue of societal accommodation. Notably, our exemplars seem to indicate that profiles of harm may change over time as societies change. Although this is seen in only a very small number of exemplars, this means that we cannot provide future decision-makers with algorithmic advice about what proposal to accept or reject across the board. Just as the clinical criterion requires careful clinical judgment, so in the case of these disorders, decisions will require careful practical judgment, that weighs up a range of relevant considerations.

The second set of conceptual issues is those concerning the notion of ‘dysfunction’. In some medical disorders there is persuasive evidence of biological dysfunction (e.g. in progeria and in schizophrenia, neurogenetic mechanisms are causally linked to distressing and impairing symptoms). However, in many mental conditions, causal mechanisms are poorly understood, and psychobiological dysfunction is inferred on the basis of crude markers such as the severity of symptoms and the extent of associated distress and impairment (e.g. in mild cognitive impairment and in social anxiety disorder). Furthermore, our exemplars point to additional considerations.

In particular, in some cases of putative mental disorder, even though there are symptoms, as well as associated distress and impairment, there are still reasons to doubt the presence of underlying psychobiological dysfunction. First, the symptoms may simply reflect apparently normal processes, such as memory loss with age, or bereavement symptoms after a loss. Second, the symptoms may represent an understandable response to particular circumstances, other than those in Table 3, criterion C. Suicidal ideation, for example, may be reasonable under certain circumstances. Thus, judgments about dysfunction, again, require careful practical judgment, weighing up a range of relevant considerations.

While the concept of ‘dysfunction’ is a useful one for conceptualizing mental disorders, when new entities are proposed in the future, it would be ideal to have more sharply defined indicators of dysfunction. Symptom severity, excessiveness, and duration may be helpful in indexing dysfunction (e.g. pointing to hypersexuality, or obsessional jealousy), but they are rough indicators that run the risk of relying on a statistical definition of dysfunction. At the same time, it is notable how rarely molecular evidence, *per se*, is able to index dysfunction; *crucially, biological difference does not point to dysfunction*.

The third set of conceptual issues relates to the type and extent of data required to reach conclusions about harm and dysfunction. In our proposed DSM-5 definition of mental disorder, we emphasized the importance of evidence for diagnostic validity and clinical utility. Diagnostic validity is supported, in part, by data that point to the involvement of specific etiological mechanisms; such data support assertions that psychobiological dysfunction is present and can be addressed by health interventions. Clinical utility is supported, in part, by data indicating that clinical assessment and intervention will be helpful; such data support assertions that harm is present and can be diminished. These issues are not listed in the DSM-5 text defining mental disorders, but our exemplars suggest that they are useful considerations.

Thus, across different proposals for disorders, there have been differences in the type and extent of data that support diagnostic validity and clinical utility. This is apparent in discussions of behavioral addictions, APS, and simple type schizophrenia. In behavioral addictions, some entities (e.g. gambling) have a great deal of data supporting diagnostic validity and clinical utility, while others (e.g. gaming) have fewer supporting data. In the case of simple type schizophrenia there are insufficient data to demonstrate diagnostic validity, and in the case of APS, there are insufficient data to demonstrate clinical utility.

It is notable that most discussions of the definition of mental disorders focus on conceptual issues and are therefore quite different from a data-oriented approach to the validation of entities, once they are considered to be disorders. It may be useful to incorporate explicitly the importance of a validation-oriented approach into conceptual discussions. Some in the field expect that once internet gaming gathers more high-quality validity and utility data, it too will be accepted as a disorder. Our view is that the field should recognize the potential importance of evidence of diagnostic validity and clinical utility in the definition of a mental disorder, and that future revisions further clarify the type and extent of data needed to support such judgments.

In summary, this paper has taken an exemplar-based approach to the question of defining mental disorders. We had hoped to extract a set of practical guidelines that future nosologists could draw on when discussing proposals for new entities. The conceptual issues that emerge from our exemplars are, however, complex, indicating that any future proposal will need to be assessed on its particular merits, using practical judgment. Nevertheless, several proposals for the field emerged. First, while harm is useful for defining mental disorder, some proposed entities may require careful consideration of individual *v.* societal harm, as well as of societal accommodation. Second, while dysfunction is useful for conceptualizing mental disorders, the field would benefit from developing more sharply defined indicators of dysfunction. Third, it would be useful to incorporate evidence of diagnostic validity and clinical utility into the definition of a mental disorder and to further clarify the type and extent of data needed to support such judgments.
